# Validation of a Novel Modified Aptamer-Based Array Proteomic Platform in Patients with End-Stage Renal Disease

**DOI:** 10.3390/diagnostics8040071

**Published:** 2018-10-08

**Authors:** Zhongji Han, Zhousheng Xiao, Kamyar Kalantar-Zadeh, Hamid Moradi, Tariq Shafi, Sushrut S. Waikar, L. Darryl Quarles, Zhi Yu, Adrienne Tin, Josef Coresh, Csaba P. Kovesdy

**Affiliations:** 1 Department of Medicine-Nephrology, University of Tennessee Health Science Center, Memphis, TN 38103, USA; zhan10@uthsc.edu (Z.H.); zxiao2@uthsc.edu (Z.X.); dquarles@uthsc.edu (L.D.Q.); 2 Department of Medicine, University of California, Irvine, CA 92868, USA; kkz@uci.edu (K.K.-Z.); hmoradi@uci.edu (H.M.); 3 Department of Epidemiology, Biostatistics and Medicine, Johns Hopkins Medical Institutions, Baltimore, MD 21287, USA; tshafi@jhmi.edu; 4 Division of Renal Medicine, Brigham and Women’s Hospital, Harvard Medical School, Boston, MA 02115, USA; swaikar@bwh.harvard.edu; 5 Johns Hopkins Bloomberg School of Public Health Welch Center for Prevention, Epidemiology, and Clinical Research, Baltimore, MD 21205, USA; zyu33@jhmi.edu (Z.Y.); atin1@jhu.edu (A.T.); coresh@jhu.edu (J.C.); 6 Nephrology Section, Memphis VA Medical Center, Memphis, TN 38104, USA

**Keywords:** end-stage renal disease, SOMAscan, validation, biomarker

## Abstract

End stage renal disease (ESRD) is characterized by complex metabolic abnormalities, yet the clinical relevance of specific biomarkers remains unclear. The development of multiplex diagnostic platforms is creating opportunities to develop novel diagnostic and therapeutic approaches. SOMAscan is an innovative multiplex proteomic platform which can measure >1300 proteins. In the present study, we performed SOMAscan analysis of plasma samples and validated the measurements by comparison with selected biomarkers. We compared concentrations of SOMAscan-measured prostate specific antigen (PSA) between males and females, and validated SOMAscan concentrations of fibroblast growth factor 23 (FGF23), FGF receptor 1 (FGFR1), and FGFR4 using Enzyme-Linked immunosorbent assay (ELISA). The median (25th and 75th percentile) SOMAscan PSA level in males and females was 4304.7 (1815.4 to 7259.5) and 547.8 (521.8 to 993.4) relative fluorescence units (*p* = 0.002), respectively, suggesting biological plausibility. Pearson correlation between SOMAscan and ELISA was high for FGF23 (*R* = 0.95, *p* < 0.001) and FGFR4 (*R* = 0.69, *p* < 0.001), indicating significant positive correlation, while a weak correlation was found for FGFR1 (*R* = 0.13, *p* = 0.16). In conclusion, there is a good to near-perfect correlation between SOMAscan and standard immunoassays for FGF23 and FGFR4, but not for FGFR1. This technology may be useful to simultaneously measure a large number of plasma proteins in ESRD, and identify clinically important prognostic markers to predict outcomes.

## 1. Introduction

Chronic kidney disease (CKD) represents an important public health problem, affecting 11.1% of the population worldwide [1]. End-stage renal disease (ESRD), which is defined as a glomerular filtration rate (GFR) of less than 15 mL/min/1.73 m^2^ or receiving renal replacement therapy, is also showing an increasing prevalence: in the US alone there were 57,420, 391,121 and 678,383 prevalent ESRD patients in 1980, 2000 and 2014, respectively [2]. Despite significant amounts of resources being spent on technological research and clinical trials, mortality rates in patients with ESRD remain approximately 5-fold higher than the age- and gender-adjusted mortality of general Medicare patients in all age groups [2]. New approaches are needed to identify potential treatment targets which might have beneficial effects on these patients’ survival.

Patients with ESRD suffer from a high burden of morbidity and mortality, in large part related to cardiovascular disease complications, but also from other causes such as infections [3,4]. This high burden of morbidity and mortality cannot be fully explained by current prevailing risk factor paradigms, such as hypercholesterolemia, hypertension, or diabetes mellitus. Several previous studies have shown that classical associations and risk profiles, which are very well established in the general population, cannot be detected in hemodialysis patients [5,6]. A number of non-traditional risk factors have been proposed to explain the high morbidity and mortality observed in ESRD, such as inflammation, oxidative stress, mineral and bone disorders, anemia, other yet-to-be-identified metabolic abnormalities linked to the uremic state, or a combination of these [7,8,9,10,11,12]. Particularly, fibroblast growth factor 23 (FGF23) has emerged as one of the most powerful predictors of adverse outcomes in patients with CKD and ESRD. FGF23 is a hormone produced by osteoblasts and osteocytes in bone that acts on the bone-kidney endocrine network to regulate phosphate and vitamin D metabolism. FGF receptor 1 (FGFR1) or FGFR4 can form complexes with α-Klotho to comprise a functional FGF23 receptor [13,14].

There are currently no approved therapeutic interventions known to reduce mortality in patients with ESRD. It is thus necessary to identify novel biomarkers which can assist the accurate and early diagnosis of risk factors and conditions relevant to ESRD-associated mortality, thereby allowing clinicians to tailor their therapy for this particular population of patients. Commonly used biomarker screening techniques have their limitations. For example, many challenges remain for mass spectrometry (MS) in clinical proteomics, including issues of sensitivity, specificity, reproducibility, validation, and cost [15]; while antibody-based technology, such as Enzyme-Linked immunosorbent assay (ELISA), is sensitive, and it cannot be multiplexed above a few dozens of simultaneous measurements [16].

Proteomic array platforms have been developed to improve diagnostics for conditions with large unmet clinical needs, such as oncology, renal disease, and infections. These diseases would benefit greatly from early detection and diagnosis, which could be aided by multiplex proteomic assays [17,18,19,20,21]. Recently, a modified aptamer-based technology, SOMAscan, was developed as a highly sensitive and multiplexed proteomics platform [22,23,24]. SOMAscan is based on Slow Off-rate Modified Aptamers (SOMAmers) that recognize specific conformational epitopes of natural 3D proteins with high specificity and sensitivity [25]. The SOMAscan platform is a versatile and powerful tool that allows the large-scale comparison of proteome profiles within discrete subpopulations. The SOMAscan assay measures levels of 1317 analytes using only 65 µL of complex biological fluids over a wide dynamic range.

Due to the novel nature of the technology used by SOMAscan, its large scale practical implementation necessitates the independent validation of its measurements against those done with standard accepted methods such as ELISA. This is especially important in populations such as ESRD, which suffer from complex metabolic abnormalities affecting scores of biomarkers, the concentrations of which are often several magnitudes higher compared to normal physiologic ranges. In the present study, we used the SOMAscan platform for the investigation of the plasma proteome associated with ESRD using 21 ESRD patients’ plasma who received maintenance hemodialysis, and validated the SOMAscan measurements of select proteins (FGF23, FGFR1, and FGFR4) against those performed using standard methods.

## 2. Materials and Methods

### 2.1. Study Population and Samples

After providing informed consent, 4023 patients who received maintenance hemodialysis from US-wide dialysis units within a large dialysis organization (DaVita, Inc., Denver, CO, USA) were prospectively enrolled during 2011–2013, and underwent specimen collections (including plasma, serum, and whole blood) on a quarterly basis for up to one year. Following completion of the specimen collection phase, the biospecimens and patients’ corresponding de-identified clinical and outcome information was allocated to four academic centers (University of Tennessee Health Science Center (UTHSC), University of California Irvine, Harvard University, and the Johns Hopkins Hospital) for further investigations. For the present study we used plasma samples from 21 ESRD patients randomly selected from the repository housed at UTHSC (UT-DaVita Hemodialysis Biorepository, Memphis, TN, USA). The study was approved by the Institutional Review Board (IRB) of the University of Tennessee Health Science Center [UTHSC, IRB protocol numbers 16-04357-XP (approved: 24 January 2016) and 17-05299-XP (approved: 19 May 2017)].

### 2.2. SOMAscan Assay

Human plasma samples were analyzed using a SOMAmer-based capture array called “SOMAscan” (SomaLogic, Inc., Boulder, CO, USA). A SOMAscan Satellite Site at Washington University in St. Louis (St. Louis, MO, USA) performed all proteomic assessments, which were blinded to the clinical characteristics of enrolled patients for this study. This assay was performed as described previously [26,27]. Briefly, plasma samples were incubated with SOMAmer reagents. After washing, the protein analytes bound to SOMAmer reagents were labeled with biotin. The SOMAmer-protein complexes were then incubated with streptavidin-coated beads. Under denaturing conditions, SOMAmer reagents were detached from the SOMAmer-protein complexes, and SOMAmer reagents were then collected and hybridized to complementary sequences on a microarray. SOMAmers are modified nucleic acid aptamers, each with both unique protein binding characteristics and a unique identifying primary nucleic acid sequence that can be detected and quantified by DNA microarray [25]. Therefore, all SOMAscan measures are reported as relative fluorescence units (RFU). Each SOMAmer has been validated for its specificity, upper and lower limits of detection, and intra- and inter- assay variability.

The SOMAscan assay utilized for our study measures 1317 proteins simultaneously in a small volume (65 µL) of plasma. The list of the 1317 proteins examined in this study is presented with their UniProt IDs and Gene IDs in Table S1. Plasma dilutions (0.005%, 1% and 40%) were applied to capture low-, medium-, and high-abundant proteins. Each of the proteins measured in plasma by the version of the SOMAscan assay performed in this study has its own target SOMAmer reagent. SOMAmer reagents were selected for 1317 human proteins (secreted proteins, extracellular domains, and intracellular proteins) that belong to broad biological groups, including receptors, kinases, cytokines, proteases, growth factors, protease inhibitors, hormones, and structural proteins [25,26]. Most of these proteins are involved in signal transduction pathways, stress response, immune processes, phosphorylation, proteolysis, cell adhesion, cell differentiation, and intracellular transport.

### 2.3. Quality Control Assessment

According to the SOMAscan version 3.2 assay data quality-control procedures, hybridization control normalization, median signal normalization, and calibration normalization were employed to remove the assay and sample bias [22,25,28]. The quality control was performed at the sample and SOMAmer level, and involves the use of control SOMAmers on the microarray and calibration samples. Each plate design includes a buffer well (no samples added), 2 quality control samples, and 5 calibrator samples provided by Somalogic. Quality control and calibrator samples are pooled samples composed of the same matrix as the plasma samples being measured in the plate. The purpose of these samples is to assess the quality of measurements obtained from one single plate. The hybridization control normalization removes any variability from sample-to-sample that may have been added from the end of the SOMAscan assay through microarray scanning, including any scanner intensity differences, so hybridization control normalization is the source of sample-to-sample variability. The median signal normalization uses all the SOMAmer signals on a given subarray to remove sample or assay biases that may be due to differences between samples in overall protein concentration, pipetting variation, variation in reagent concentrations, assay timing, and any other source of systematic variability within a single plate run. The calibration normalization is necessary for the correction of run-to-run variations and is performed during each run. It is achieved using common pooled calibrator plasma samples that have been run in replicate numerous times at the manufacturer (Somalogic, Boulder, CO, USA), to create an external reference or a set of standard values. According to Somalogic, with the quality control assessment, a single assay was used per plasma sample, and thus, no technical replicates were performed. The criteria for the hybridization control and median signal normalization scale factors should be between 0.4 and 2.5; the median of the calibration scale factor should be within 1.0 ± 0.2, and a minimum of 95% of individual SOMAmer must have a calibration scale factor within the median ±0.4.

### 2.4. ELISA Assays

Selected candidate proteins were validated using sandwich enzyme immunoassays. Plasma full-length FGF23 level was measured using the FGF23 ELISA kit (Kainos Laboratories, Tokyo, Japan) following the manufacturer’s recommendations. Plasma FGFR1 and FGFR4 levels were measured using the FGFR1 ELISA kit (Human, Catalog number: OKEH00124, Aviva Systems Biology, San Diego, CA, USA) and the FGFR4 ELISA kit (Human, Catalog number: OKDD00266, Aviva Systems Biology, San Diego, CA, USA) following the manufacturer’s recommendations. The plasma was diluted to fall within the linear range of each respective assay.

### 2.5. Statistical Analysis

SOMAscan proteomic data were reported in relative fluorescence units (RFU). Quantile normalization and log-transformation were performed for all RFU-reported data. Principal component analysis was performed to assess for the presence of plate effect. Student’s *t* tests and Wilcoxon’s rank sum tests were used to compare continuous and categorical variables, respectively. Correlations between the SOMAscan and ELISA measurements were assessed using Pearson correlation coefficients using the transformed values of the raw values. Analyses were performed using R (https://www.rstudio.com/products/rpackages/) and Stata MP 15 (www. Stata.com).

## 3. Results

### 3.1. Study Subjects and Sample Quality Assessment

Patients were 57.0 ± 13.7 years old, 52% were male, 48% were African American, and the median Charlson comorbidity index score (range) was 6 (2 to 8) (Table 1). The SOMAscan run included two buffer controls, four quality controls, and ten pooled calibrator plasma samples. Sample data was first normalized to remove hybridization variation within a run, followed by median normalization across all samples to remove other assay biases within the run, and finally calibrated to remove assay differences between runs. All 21 samples passed the quality control assessment (Table S2). No plate effect was detected by principal component analysis (Figure S1), and 1272 of 1317 proteins passed the quality control assessment.

### 3.2. SOMAscan Results for Prostate Specific Antigen

**Figure 1:**
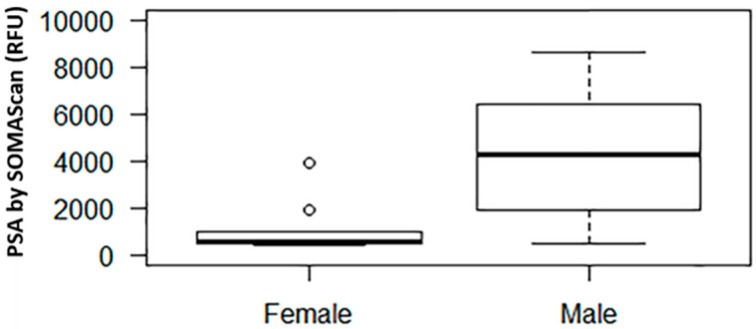
Relative fluorescence unit (RFU) signal of prostate specific antigen (PSA) by SOMAscan in males vs. females. The middle horizontal line represents the median. The box bounds the 25th and 75th percentile of the points. The dotted lines connect to the thin horizontal line denoting 1.5 interquartile range (IQR) from the median. The dots denote points with values outside of the 1.5 IQR from the median.

In our SOMAscan assay, among males and females the median (interquartile range, IQR) prostate specific antigen (PSA) signal level was 4304.7 (1815.4 to 7259.5) and 547.8 (521.8 to 993.4) RFU, respectively (*p* = 0.002, Figure 1). For reference, the mean (±SD) RFU value of the negative control was 279.7 ± 26.8.

### 3.3. SOMAscan and ELISA Results for FGF23, FGFR4, and FGFR1

**Figure 2:**
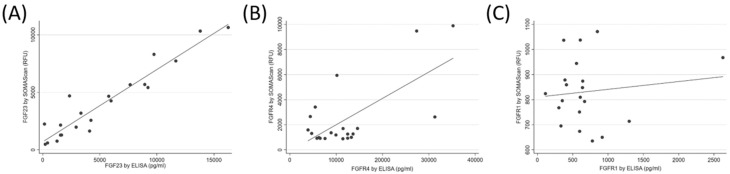
Correlation between SOMAscan and Enzyme-Linked immunosorbent assay (ELISA) measurements of fibroblast growth factor 23 (FGF23, panel (**A**)), fibroblast growth factor receptor 4 (FGFR4, panel (**B**)), and fibroblast growth factor receptor 1 (FGFR1, panel (**C**)) in human plasma.

The median (IQR) FGF23 SOMAscan signal was 3194.1 (1463.0 to 5677.7) RFU, and the median (IQR) FGF23 concentration by ELISA was 4106.2 (1562.3 to 9078.5) pg/mL. The SOMAscan FGF23 levels correlated well with the ELISA measurements of FGF23 (*R* = 0.95, *p* < 0.001, Figure 2A). The median (IQR) FGFR4 SOMAscan signal was 1308.6 (954.0.0 to 2637.1) RFU, and the median (IQR) FGFR4 concentration by ELISA was 10,171.7 (6075.8 to 13,467.5) pg/mL. There was a strong correlation between the SOMAscan and the ELISA-measured FGFR4 levels (*R* = 0.68, *p* < 0.001, Figure 2B). The median (IQR) FGFR1 SOMAscan signal was 824.1 (732.4 to 955.9) RFU, and the median (IQR) FGFR1 concentration by ELISA was 602.8 (381.7 to 723.6) pg/mL. Only a weak correlation was found between the FGFR1 SOMAscan results and the ELISA measurements (*R* = 0.13, *p* = 0.16, Figure 2C). For reference, the mean (±SD) RFU value of the negative control for FGF23, FGFR4, and FGFR1 was 322.4 ± 10.6, 1153.7 ± 58.7, and 407.4 ± 30.7, respectively.

## 4. Discussion

We performed quality control assessment, biological plausibility confirmation, and validation of the SOMAscan proteomic platform on plasma samples when applied to patients with ESRD. We examined select biomarkers of bone and mineral metabolism which are known to display marked elevations in patients with ESRD (compared to individuals with normal kidney function) to determine the validity of SOMAscan measurements in this population. Our results suggest that the SOMAscan platform can be informative of the plasma proteome of ESRD patients, confirming earlier validation studies performed in patients with normal kidney function [29].

SOMAscan has become an important platform for diagnostic and prognostic biomarker identification. For example, De Groote et al. identified and validated a six-marker signature for the diagnosis of active pulmonary tuberculosis using the SOMAscan assay on 1470 serum samples from seven countries where tuberculosis is endemic [30]. Ostroff et al. also reported a large-scale clinical application of the SOMAscan platform [31]. They identified 44 candidate biomarkers, and developed a 12-protein panel that discriminates non-small cell lung cancer from controls with 91% sensitivity and 84% specificity in cross-validated training, and 89% sensitivity and 83% specificity in a separate verification set, with similar performance for early and late stage non-small cell lung cancer. Based on this study, a clinical blood test to enable an earlier diagnosis of lung cancer is being developed. In addition, an earlier version of SOMAscan was applied to a clinical study of 42 patients with non-dialysis dependent CKD, and it identified 58 potential novel biomarkers of CKD [26]. However, the novelty of such technologies necessitates that they are independently validated against traditional laboratory methods to assure their reliability.

The biological validity of the SOMAscan measurement can be evaluated by examining the concentration of biomarkers that are expected to show significant differences in certain groups, such as in males versus females. PSA is released primarily from the epithelial cells of the prostate gland and is thus expected to have substantially higher concentrations in males [32], although PSA has also been shown to be expressed in various tissues in females [33], and hence it should be detectable at lower circulating levels. As expected, the PSA level among males was significantly higher than the PSA level among females, suggesting biological plausibility. We also validated the SOMAscan measurement of three proteins against commercial ELISA assays. Validation is the most important and difficult step for high multiplex biomarker discovery. We selected FGF23 and its receptors, FGFR1 and FGFR4, as the candidate proteins for validation. FGF23 levels are markedly elevated in ESRD, and FGF23 was recognized as a powerful predictor of adverse outcomes in this patient population [13,14]. Among the examined proteins, we found strong correlations for FGF23 and FGFR4, while the measurements for FGFR1 were only weakly correlated. The correlation discrepancy between FGFR4 and FGFR1 could be due to multiple circulating isoforms of FGFR1 and the single isoform of FGFR4 existing in plasma samples [34,35]; such a lack of correlation was also reported by other studies [36,37], and it is most likely related to the different epitope recognition sites between SOMAmers and antibodies for ELISA. Cross-reactivity and negative cooperative binding could also be responsible for the lack of agreement. Further delineation of specificity of the aptamers is needed for better validation.

Our study is the first to formally examine the validity of SOMAscan in patients with ESRD, yet it has limitations that need to be considered when interpreting its findings. We only examined 21 patients, which may have precluded the detection of weaker correlations, and which also precludes more in-depth analyses such as sex- and race-associated differences. We examined only a small number of proteins, which limits our ability to provide a wide-ranging endorsement (or lack thereof) of SOMAscan in ESRD. Further studies may be needed to examine the validity of SOMAscan for measurement of other circulating proteins known to have abnormal values in ESRD. We used ELISA as the standard to compare against SOMAscan, since it is the method most often used in practice to measure the proteins in question. However, ELISA cannot be regarded as a true gold standard, due to problems with its standardizations and concerns about commercially available kits [38].

## 5. Conclusions

We evaluated the reliability of SOMAscan assay through quality control assessment, biological plausibility, and validation. We describe a good but not perfect correlation between the SOMAscan assay and commercially available immunoassays with selected proteins. Despite the small number of examined patients, this pilot study provides new evidence for the utility of the SOMAscan platform for high multiplex proteomics analysis in patients with ESRD, and suggests that this platform could be used to identify novel and specific biomarkers in this patient population.

## Figures and Tables

**Table 1:** Characteristics of participants.

Characteristics	All (*N* = 21)	Female (*N* = 10)	Male (*N* = 11)
Age	57.0 (13.7)	57.7 (10.3)	56.3 (16.9)
Race			
African American	10 (47.6)	5 (50.0)	5 (45.5)
Caucasian	7 (33.3)	3 (30.0)	4 (36.4)
Hispanic	4 (19.1)	2 (20.0)	2 (18.2)
Diabetes mellitus	14 (66.7)	8 (80.0)	6 (54.6)
Charlson comorbidity index	5.0 ± 1.9	5.1 ± 1.4	4.9 ± 2.3

## Supplementary Materials

The following are available online at http://www.mdpi.com/2075-4418/8/4/71/s1. Figure S1: Principal component analysis of plate effect. Table S1: List of 1317 proteins examined using SOMAscan with SOMAmer SeqID, UniProt ID, and Gene ID. Table S2: Quality control assessment results for 21 samples. All 21 samples passed the quality control assessment.
