# Supplementary figures and images for: Validation of a Novel Modified Aptamer-Based Array Proteomic Platform in Patients with End-Stage Renal Disease
Source: Diagnostics (Basel). 2018 Oct 8;8(4):71. doi: 10.3390/diagnostics8040071 (PMC6316431; doi:10.3390/diagnostics8040071)

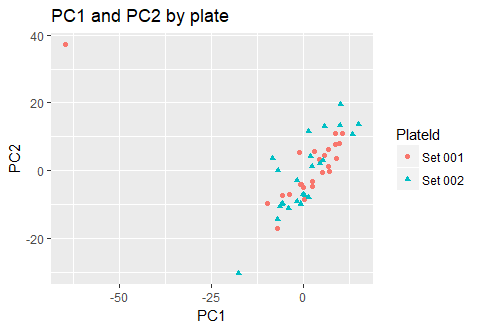

Supplement: Supplementary file 1 [file diagnostics-08-00071-s001.zip › Supplementary Figure 1.xlsx.tif]
